# Supplementary material for: Predictors and trends of Caesarean section and breastfeeding in the Eastern Mediterranean region: Data from the cross-sectional Cyprus Women’s Health Research (COHERE) Initiative
Source: PLoS One. 2023 Jul 7;18(7):e0287469. doi: 10.1371/journal.pone.0287469 (PMC10328327; doi:10.1371/journal.pone.0287469)
Supplement: S1 Table — (DOCX) [file pone.0287469.s001.docx]

**Supplementary Table 1. Demographic characteristics and comparison between women included and excluded from the study due to missing data**

|  | **Excluded from the study (data missing)** | **Included in the study (no data missing)** | **p-value** |
| --- | --- | --- | --- |
|  |  |  |  |
| **Year of pregnancy** |  |  | 0.083 |
| <1995 | 164 (26.2) | 631 (22.3) |  |
| 1995-2005 | 193 (30.8) | 961 (33.9) |  |
| >2005 | 270 (43.1) | 1,244 (43.9) |  |
|  |  |  |  |
| **Age at pregnancy** |  |  | 0.301 |
| <25 | 391 (51.5) | 1,355 (47.8) |  |
| 25-29 | 262 (34.5) | 1,051 (37.5) |  |
| 30-34 | 89 (11.7) | 351 (12.4) |  |
| 35+ | 17 (2.2) | 79 (2.8) |  |
|  |  |  |  |
| **Ethnicity** |  |  | 0.484 |
| Turkish Cypriot | 464 (70.7) | 2,037 (71.8) |  |
| Turkish | 162 (24.7) | 648 (22.9) |  |
| Mixed/Other | 30 (4.6) | 151 (5.3) |  |
|  |  |  |  |
| **Education** |  |  | **0.003** |
| Primary/Middle school | 110 (18.3) | 448 (15.8) |  |
| High school/Post-secondary | 248 (41.2) | 1,005 (35.4) |  |
| Undergraduate degree | 170 (28.2) | 946 (33.4) |  |
| Postgraduate degree | 74 (12.3) | 437 (15.4) |  |
|  |  |  |  |
| **Residence type** |  |  | 0.248 |
| Village | 457 (57.6) | 1,567 (55.3) |  |
| City | 337 (42.4) | 1,269 (44.8) |  |
|  |  |  |  |
| **Pre-term birth** |  |  | 0.458 |
| No | 736 (92.7) | 2,650 (93.4) |  |
| Yes | 58 (7.3) | 186 (6.6) |  |
|  |  |  |  |
| **IVF treatment** |  |  | 0.807 |
| No | 536 (95.0) | 2,702 (95.3) |  |
| Yes | 28 (5.0) | 134 (4.7) |  |
|  |  |  |  |
| **Baby weight (kg)** |  |  | 0.482 |
| Normal (≥2.5kg - <4.0kg) | 436 (82.9) | 2,409 (84.9) |  |
| Under (<2.5kg) | 41 (7.8) | 198 (7.0) |  |
| Over (≥4.0kg) | 49 (9.3) | 229 (8.1) |  |
